# De Novo Gene Variants and Familial Bipolar Disorder

**DOI:** 10.1001/jamanetworkopen.2020.3382

**Published:** 2020-05-08

**Authors:** Claudio Toma, Alex D. Shaw, Bronwyn J. Overs, Philip B. Mitchell, Peter R. Schofield, Antony A. Cooper, Janice M. Fullerton

**Affiliations:** 1 Neuroscience Research Australia, Sydney, New South Wales, Australia; 2 School of Medical Sciences, University of New South Wales, Sydney, New South Wales, Australia; 3 School of Psychiatry, University of New South Wales, Sydney, New South Wales, Australia; 4 Black Dog Institute, Prince of Wales Hospital, Sydney, New South Wales, Australia; 5 Garvan Institute of Medical Research, Sydney, New South Wales, Australia; 6 St Vincent’s Clinical School, University of New South Wales, Sydney, New South Wales, Australia

## Abstract

This case-control study explores de novo candidate gene variants in 18 multiplex families with bipolar disorder.

## Introduction

The detection of de novo variants (DNVs) by next-generation sequencing has facilitated the identification of candidate genes in psychiatric disorders.^1,2^ Spontaneous DNV mutations are estimated to explain approximately 5% of genetic liability in autism and schizophrenia.^1,2^ In bipolar disorder (BD), common genetic variants explain approximately 30% of the heritability,^3^ and rare inherited variants also contribute to disease risk.^4^ However, the involvement of DNVs in BD is largely unexplored, with only 1 independent study^5^ reporting 71 DNVs from 79 singleton BD families. We present a DNV study in 18 multiplex bipolar families, combining 32 individuals previously reported^4^ with 29 additional participants.

## Methods

This study was approved by the University of New South Wales Ethics Committee. Participants provided written consent. This study follows the Strengthening the Reporting of Genetic Association Studies (STREGA) reporting guideline.

We performed whole-genome sequencing of 9 multiplex families (47 participants, including 29 offspring; there were 8 unaffected offspring and 21 case offspring with BD type I, BD type II, or schizoaffective-disorder bipolar type) in this case-control study. Identification of DNVs used 2 variant calling software packages, GATK version 3.4.0 (Broad Institute) and RTG-Core sequencing software version 3.7.1 (Real Time Genomics Ltd). All variants were validated by Sanger sequencing and were combined with variants previously identified by whole-exome sequencing in 32 offspring,^4^ for a final sample of 18 multiplex families (99 individuals, including 61 offspring; there were 43 cases and 18 unaffected offspring individuals). The DNA samples were prepared and whole-genome sequencing was performed on the HiSeq X platform at The Kinghorn Centre for Clinical Genomics, Garvan Institute of Medical Research, Sydney, Australia. Whole-exome sequencing was previously performed at the Lottery State Biomedical Genomics Facility, University of Western Australia.

For each gene harboring DNVs, a gene-based association study was performed using the *multi snp-wise* model in MAGMA statistical software version 1.06b (VU University, Amsterdam, The Netherlands) using a threshold of *P *<* *.05 and genome-wide association study summary statistics from the Psychiatric Genomics Consortium. For BD, there were 20 352 cases and 31 358 controls^3^; for schizophrenia, there were 40 675 cases and 64 643 controls; and for major depression, there were 170 756 cases and 329 443 controls. Protein interaction network analysis was performed using IPA statistical software release date February 8, 2019 (Qiagen Digital Insights). Data were analyzed from February 2017 to June 2019.

## Results

Thirty-two DNVs from 12 female and 17 male offspring were validated and combined with 31 DNVs from our previous report,^4^ for a combined analysis of 63 coding DNVs from 61 white participants (30 male participants [50%]; mean [SD] age, 38.4 [8.5] years). In total, 42 DNVs were identified in cases (Table) and 21 variants were identified in unaffected offspring. The overall DNV mutation rate was no different between cases and unaffected relatives (42 of 43 [0.98] and 21 of 18 [1.17], respectively; *P* = .65, 2-tailed χ^2^ statistic). However, missense and gene-truncating DNVs were statistically significantly more frequent in cases compared with unaffected relatives (32 of 42 [75%] vs 10 of 21 [47%]; *P* = .01, 1-tailed test of proportions). Interestingly, the rate for potentially disrupting DNVs in brain-expressed genes (missense, classified as pathogenic in both SIFT statistical software version 5.0.2 [Bioinformatics Institute] and PolyPhen-2 statistical software version 2.2.2 [Harvard Medical School], and nonsense and gene-truncating indels)^1,4^ was higher in participants with BD compared with unaffected relatives (14 of 42 [33%] vs 3 of 21 [14%]), which is comparable to the previous BD report (30%)^5^ and rates observed in autism (36%) and schizophrenia (34%).^2^

**Table.** List of 42 De Novo Variants Identified in 43 Affected Offspring From 18 Multiplex Families With BD

Chromosome: position^a^	Reference allele/alternative allele	Phenotype	Gene	Change^b^	Brain expression^c^	*P* value^d^		
						BD	SCZ	MDD
16:2 350 069	G/A	SZMA	*ABCA3*	N (syn)	1	.006^e^	.44	.37
21:43 704 788^f^	G/A	SZMA	*ABCG1*	D/N (mis) D-P	1	.04^e^	.90	.94
19:41 860 693^f^	T/C	SZMA	*B9D2*	Y/C (mis) T-B	1	.60	.72	.15
8:22 064 885	C/TTTCGGCACCACC	BDI	*BMP1*	R/X (stop)	1	.09	.57	.58
17:4 803 420^f^	TTGTTAGAG/CAGTACC	BDI	*C17orf107*	F/X (fs)	1	.67	.57	.79
19:13 885 321	G/A	SZMA	*C19orf53*	R/H (mis) D-U	1	.16	.046^e^	.41
2:175 677 196^f^	C/T	BDI	*CHN1*	V/I (mis) D-P	1	.67	.03^e^	.82
17:42 828 028^f^	C/T	SZMA	*DBF4B*	P/S (mis) D-B	0	.39	.006^e^	.21
3:98 536 707	T/A	BDI	*DCBLD2*	E/V (mis) D-P	1	.72	.30	.08
13:36 699 938^f^	C/A	BDI	*DCLK1*	G/W (mis) D-P	1	.62	.21	.07
1:20 980 731	G/A	SZMA	*DDOST*	A/V (mis) T-B	1	.60	.85	.61
4:99 807 668^f^	C/T	SZMA	*EIF4E*	L (syn)	1	.13	.54	.16
5:79 809 539	C/T	BDI	*FAM151B*	L (syn)	0	.28	.46	.49
15:83 447 636	G/A	BDI	*FSD2*	L (syn)	0	.30	2.75 × 10^−5^^e^	.03^e^
2:155 099 380^f^	A/T	SZMA	*GALNT13*	G (syn)	1	.13	.002^e^	.02^e^
16:72 094 659	TGGA/T	BDI	*HP*	LE/L (inf_del)	1	.005^e^	.02^e^	.047^e^
6:150 719 321^f^	C/T	BDI	*IYD*	T/M (mis) T-B	0	.20	.18	.60
3:47 958 042	T/C	SZMA	*MAP4*	I/M (mis) D-P	1	.07	.04^e^	.10
6:36 944 299^f^	C/T	BDI	*MTCH1*	A/T (mis) T-B	1	.68	.33	.52
10:5 498 065	C/G	SZMA	*NET1*	L/V (mis) D-P	1	.90	.29	.41
11:6 789 509	C/T	BDII	*OR2AG2*	R/H (mis) T-B	0	.96	.23	.35
11:66 633 693	C/T	BDI	*PC*	A/T (mis) T-P	1	7.61 × 10^−7^^e^	.01^e^	.02^e^
5:140 870 484	T/C	BDII	*PCDHGC5*	D (syn)	1	.06	.002^e^	.37
11:3 838 696	C/T	BDI	*PGAP2*	S/L (mis) T-P	1	.36	.74	.48
1:114 253 066	C/G	BDI	*PHTF1*	R/P (mis) T-P	1	.01^e^	.82	.42
10:124 187 853	A/G	BDII	*PLEKHA1*	E/G (mis) T-B	1	.21	.006^e^	.92
14:88 946 537^f^	G/A	RUD	*PTPN21*	S/L (mis) D-P	1	.43	.28	.002^e^
19:5 976 538^f^	C/A	SZMA	*RANBP3*	V/L (mis) D-B	1	.32	.28	.54
12:53 509 433^f^	G/C	BDI	*SOAT2*	E/Q (mis) D-P	0	.03^e^	.27	.44
12:64 377 852	C/T	BDI	*SRGAP1*	R/W (mis) D-P	1	.20	.40	.67
2:37 193 554^f^	G/A	BDII	*STRN*	A/V (mis) T-B	1	.44	<.001^e^	.08
8:30 702 308^f^	T/C	BDI	*TEX15*	E/G (mis) T-B	0	.14	.28	.005^e^
19:59 067 616^f^	G/A	BDI	*UBE2M*	P/S (mis) T-B	1	7.94 × 10^−5^^e^	.002^e^	1.89 × 10^−5^^e^
17:74 395 538	T/C	BDI	*UBE2O*	P (syn)	1	.07	.005^e^	.62
4:96 090 394^f^	C/T	BDII	*UNC5C*	G (syn)	1	.37	.03^e^	.20
2:219 294 199	C/T	BDII	*VIL1*	L (syn)	1	.73	.13	.85
4:85 599 426^f^	C/T	BDI	*WDFY3*	R/Q (mis) T-P	1	.96	.06	.60
14:55 448 345^f^	G/T	SZMA	*WDHD1*	T/N (mis) D-P	1	.97	.02^e^	.84
1:43 148 332^f^	CG/C	SZMA	*YBX1*	G/X (fs) D-P	1	.73	.53	.25
19:57 953 362	C/T	BDI	*ZNF749*	A/V (mis) T-P	0	.48	.75	.02^e^
20:57 768 786^f^	C/G	SZMA	*ZNF831*	T (syn)	0	.28	.41	.90
20:47 887 288^f^	C/T	BDI	*ZNFX1*	R/H (mis) D-P	1	.005^e^	.004^e^	.09

Abbreviations: BD, bipolar disorder; BDI, bipolar disorder type I; BDII, bipolar disorder type II; MDD, major depressive disorder; RUD, recurrent unipolar depression; SCZ, schizophrenia; SZMA, schizoaffective disorder-manic type.

^a^ Genomic positions are indicated according to the GRCh37/hg19 assembly.

^b^ Predicted pathogenicity was assessed by SIFT statistical software version 5.0.2 (Bioinformatics Institute, Singapore) (D, deleterious; T, tolerated) and PolyPhen-2 statistical software version 2.2.2 (Harvard Medical School) (B, benign; P, probably damaging or possibly damaging) using Variant Effect Predictor annotation tool software release 98 (Ensembl Project).

^c^ Brain expression: 1 indicates genes expressed in brain, with reads per kilobase of transcript per million mapped reads greater than 1 (developmental transcriptomics RNA-sequence data are from the BrainSpan Atlas of the Developing Human Brain); 0 indicates that the gene is not brain expressed (reads per kilobase of transcript per million mapped reads <1).

^d^ *P* values are gene-based association values calculated with MAGMA statistical software version 1.06b (VU University, Amsterdam, the Netherlands).

^e^ Indicates nominal *P* values (*P* < .05) from the gene-based association tests. All de novo variants were rare in the general population (minor allele frequency <0.0001 in non-Finnish European population in gnomAD) or not observed, except for 12:53509433G/C_SOAT2 and 19:57953362C/T_ZNF749 with moderate allelic frequency (0.001 and 0.0003, respectively). A gene-based association test for BD, SCZ, and MDD was calculated using summary statistics of the PGC2 data sets.

^f^ Indicates variants identified from the whole-genome sequencing study.

Gene-based association tests for the 42 DNV-bearing genes implicated haptoglobin (*HP)* and pyruvate carboxylase (*PC)*, 2 brain-expressed genes associated across 3 psychiatric disorders that carry potentially pathogenic DNVs. The *PC* gene encodes a mitochondrial enzyme involved in glucose metabolism and neurotransmitter synthesis, which lies within one of 30 BD-associated loci recently reported,^3^ and it had the strongest gene-based association (*P* = 7.61 × 10^−7^; *z* statistic = 4.808; Cohen *d* = 0.042) (Table).

To identify relevant networks, 218 genes from BD-significant loci^3^ were combined with 112 genes carrying DNVs from the current and previous BD studies.^4,5^ Protein-protein interaction network analysis implicated 4 genes: microtubule associated protein 4 (*MAP4*), which promotes microtubule assembly and is reported to carry DNV in autism; WD repeat and HMG-box DNA binding protein 1 (*WDHD1*), a DNA replication initiation factor; eukaryotic translation initiation factor 4E (*EIF4E*), which interacts with cytoplasmic Fragile X Mental Retardation interaction protein and is implicated in autism; and striatin (*STRN*), a largely unknown calmodulin-binding protein with a potential role in dendritic Ca2^+^ signaling and striatal neuron maturation (Figure). Of these 4 genes, *MAP4* and *WDHD1* harbored predicted-pathogenic variants with nominal gene-based association in schizophrenia (Table).

**Figure.**
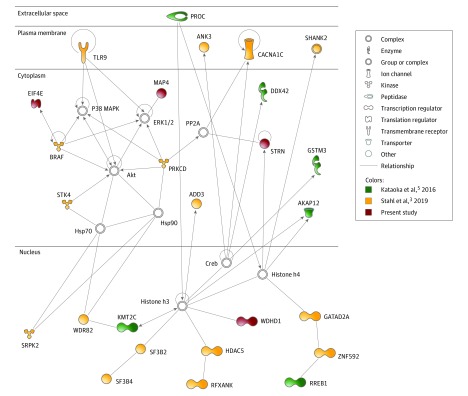
Network Showing Direct Protein-Protein Interactions Between Genes Harboring Common and De Novo Risk Variants in Bipolar Disorder (BD) Gene list was derived from 218 BD-associated genes from 30 loci identified by the genome-wide association study by Stahl et al^3^ plus 112 genes with de novo variants identified in patients with BD (70 genes from Kataoka et al^5^ and 42 genes from the present study). Only direct interactions among proteins were considered. This top network (score 41) related to hereditary disorder, neurological disease, organismal injury, and abnormalities. Upregulatory effects are represented by outward pointing arrows, downregulatory effects are represented by outward ticks, and circular arrows indicate homotypic interactions.

## Discussion

Examination of DNVs in psychiatric disorders has traditionally focused on singleton families. These findings suggest that DNVs may also contribute to mutational load in multiplex BD families, as previously observed for multiplex autism families.^6^ Although this study is limited by the small sample size, the overall de novo mutation rate was comparable in cases and unaffected offspring, whereas deleterious DNVs were observed more frequently in participants with BD, which is consistent with previous reports in autism and schizophrenia.^2^ This study highlighted *HP*, *PC*,* MAP4*, and *WDHD1* as potential susceptibility genes for BD. Additional sequencing studies in larger cohorts are needed to further delineate the impact of DNVs in BD.

## References

[1] Torrico B, Shaw AD, Mosca R, Truncating variant burden in high-functioning autism and pleiotropic effects of *LRP1* across psychiatric phenotypes. J Psychiatry Neurosci. 2019;44(5):350-359. doi:10.1503/jpn.180184 31094488 PMC6710089

[2] Fromer M, Pocklington AJ, Kavanagh DH, De novo mutations in schizophrenia implicate synaptic networks. Nature. 2014;506(7487):179-184. doi:10.1038/nature12929 24463507 PMC4237002

[3] Stahl EA, Breen G, Forstner AJ, ; eQTLGen Consortium; BIOS Consortium; Bipolar Disorder Working Group of the Psychiatric Genomics Consortium Genome-wide association study identifies 30 loci associated with bipolar disorder. Nat Genet. 2019;51(5):793-803. doi:10.1038/s41588-019-0397-8 31043756 PMC6956732

[4] Toma C, Shaw AD, Allcock RJN, An examination of multiple classes of rare variants in extended families with bipolar disorder. Transl Psychiatry. 2018;8(1):65. doi:10.1038/s41398-018-0113-y 29531218 PMC5847564

[5] Kataoka M, Matoba N, Sawada T, Exome sequencing for bipolar disorder points to roles of de novo loss-of-function and protein-altering mutations. Mol Psychiatry. 2016;21(7):885-893. doi:10.1038/mp.2016.69 27217147 PMC5414074

[6] Yuen RK, Thiruvahindrapuram B, Merico D, Whole-genome sequencing of quartet families with autism spectrum disorder. Nat Med. 2015;21(2):185-191. doi:10.1038/nm.3792 25621899

